# Tetra­kis(1,2-dimethoxy­ethane-κ^2^ *O*,*O*′)ytterbium(II) bis­(μ_2_-phenyl­selenolato-κ^2^ *Se*:*Se*)bis­[bis­(phenyl­selenolato-κ*Se*)mercurate(II)]

**DOI:** 10.1107/S1600536808019211

**Published:** 2008-07-05

**Authors:** Michael D. Romanelli, Thomas J. Emge, John G. Brennan

**Affiliations:** a Department of Chemistry and Chemical Biology, Rutgers, The State University of New Jersey, Piscataway, NJ 08854-8087, USA

## Abstract

The title salt, [Yb(C_4_H_10_O_2_)_4_][Hg_2_(C_6_H_5_Se)_6_], consists of eight-coordinate homoleptic [Yb(DME)_4_]^2+^ dications (DME is 1,2-dimethoxy­ethane) countered with [Hg_2_(SePh)_6_]^2−^ di­anions. The cations and anions have twofold rotation and inversion symmetry, respectively. The Yb centre displays a square-anti­prismatic coordination geometry and the Hg centre has a distorted tetra­hedral coordination environment. One phenyl­selenolate anion and one methyl group of a DME ligand are disordered over two positions with equal occupancies. This structure is unique in that it represents a less common mol­ecular lanthanide species in which the lanthanide ion is not directly bonded to an anionic ligand. There are no occurrences of the [Hg_2_(SePh)_6_]^2−^ dianion in the Cambridge Structural Database (Version of November 2007), but there are similar oligomeric and polymeric Hg_*x*_(SePh)_*y*_ species. The crystal structure is characterized by alternating layers of cations and anions stacked along the *c* axis.

## Related literature

For the synthesis and crystal structures of related compounds see: Berardini *et al.* (1995 ▶); Bettenhausen & Fenske (1998 ▶); Deacon *et al.* (2001 ▶); Evans *et al.* (2000 ▶); Freedman *et al.* (1997 ▶); Hyeon & Edelmann (2003 ▶); Hakansson *et al.* (1999 ▶); Kim & Kanatzidis (1991 ▶); Magull *et al.* (1991 ▶); Melman *et al.* (2002 ▶); Allen (2002 ▶); Allen *et al.* (1991 ▶).
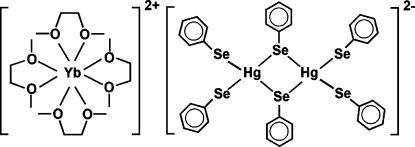


## Experimental

### 

#### Crystal data

[Yb(C_4_H_10_O_2_)_4_][Hg_2_(C_6_H_5_Se)_6_]
*M* *_r_* = 1871.06
Monoclinic, 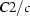

*a* = 22.688 (3) Å
*b* = 12.354 (2) Å
*c* = 22.843 (3) Å
β = 114.218 (3)°
*V* = 5839.1 (14) Å^3^
*Z* = 4
Mo *K*α radiation
μ = 10.62 mm^−1^
*T* = 100 (2) K
0.17 × 0.14 × 0.02 mm

#### Data collection

Bruker SMART APEX CCD diffractometer
Absorption correction: multi-scan (*SADABS*; Bruker, 2003 ▶) *T*_min_ = 0.18, *T*_max_ = 0.81
26008 measured reflections
5956 independent reflections
4806 reflections with *I* > 2σ(*I*)
*R*_int_ = 0.052

#### Refinement

*R*[*F* ^2^ > 2σ(*F* ^2^)] = 0.028
*wR*(*F* ^2^) = 0.064
*S* = 1.00
5956 reflections
389 parameters
734 restraints
H-atom parameters constrained
Δρ_max_ = 1.50 e Å^−3^
Δρ_min_ = −0.73 e Å^−3^

### 

Data collection: *SMART* (Bruker, 2005 ▶); cell refinement: *SAINT-Plus* (Bruker, 2003 ▶); data reduction: *SAINT-Plus*; program(s) used to solve structure: *SHELXS97* (Sheldrick, 2008 ▶); program(s) used to refine structure: *SHELXL97* (Sheldrick, 2008 ▶); molecular graphics: *SHELXTL* (Sheldrick, 2008 ▶); software used to prepare material for publication: *SHELXTL*.

## Supplementary Material

Crystal structure: contains datablocks I, global. DOI: 10.1107/S1600536808019211/rz2223sup1.cif

Structure factors: contains datablocks I. DOI: 10.1107/S1600536808019211/rz2223Isup2.hkl

Additional supplementary materials:  crystallographic information; 3D view; checkCIF report

## Figures and Tables

**Table 1:** Selected bond lengths (Å)

Yb1—O2	2.469 (4)
Yb1—O3	2.489 (3)
Yb1—O1	2.534 (4)
Yb1—O4	2.555 (4)
Hg1—Se3*B*	2.544 (3)
Hg1—Se1	2.5683 (7)
Hg1—Se3*A*	2.635 (3)
Hg1—Se2	2.6747 (6)
Hg1—Se2^ii^	2.8667 (6)

Symmetry codes: (i) 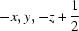; (ii) 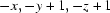.

**Fig. 1.**
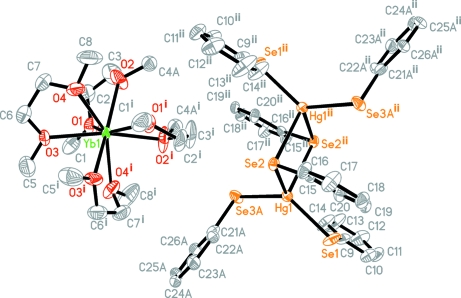
: ORTEP drawing of the title compound with thermal ellipsoids drawn at 50% probability. Hydrogen atoms are omitted for clarity. Only one component of disorder is shown. Symmetry codes: i = -x, y, 1/2 - z; ii = -x, 1 - y, 1 - z.

**Fig. 2.**
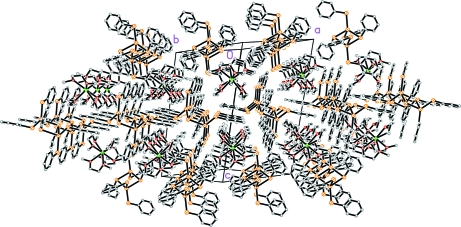
: Packing diagram of the title compound showing alternating layers of cations and anions stacking along the c direction. Hydrogen atoms are omitted for clarity. Only one component of disorder is shown.

## supplementary crystallographic information

### Comment

Lanthanide (Ln) ion salts, in which the Ln metal itself is surrounded only by neutral donor ligands and oxidized only by a non-bonded anionic molecule, make up a small portion of less common Ln complexes. It is more likely for the Ln metal to be oxidized by direct coordination of an anionic species (EPh^-^, *X*^-^, Cp^-^, *etc*.. where E = S, Se, Te, *X* = Cl, Br, I) with the metal (Hyeon *et al.*, 2003, Melman *et al.*, 2002). Similar species to the one described herein exist, such as, tris(1,2-dimethoxyethane-*O*,*O*')-diiododysprosium(II) (Evans *et al.*, 2000), rac-diiodo-tris(dimethoxyethane)samarium (Hakansson *et al.*, 1999), and tris(1,2-dimethoxyethane)-bis(isothiocyanato)ytterbium(II) (Deacon *et al.*, 2001). These structures differ from the species reported here by their having direct oxidation of the lanthanide metal with anionic coordinating ligands.

In this communication, we report the structure of a unique [Yb(DME)_4_]^2+^[Hg_2_(SePh)_6_]^2-^ molecular salt (Fig 1.). The 8-coordinate [Yb(DME)_4_]^2+^ dication in this study has approximate D-42*m* (Td) symmetry giving rise to square antiprism geometry similar to U(acac)_4_. Yb—O bonds of the dication range from 2.465 (4) Å to 2.561 (4) Å and are consistent with the range found for divalent ytterbium(II)—O*R*_2_ species, having an associated average of 2.45 Å (Cambridge Structural Database; Allen *et al.*, 1991). Consistent with the general trend, trivalent ytterbium(III)—O*R*_2_ bond distances average noticeably shorter at 2.35 Å. Like the dication, the Hg—Se bond geometries of the dianion in this study are consistent with the Hg—Se bonds found in similar oligomeric or polymeric structures (Berardini *et al.*, 1995; Bettenhausen *et al.*, 1998; Freedman *et al.*, 1997; Kim *et al.*, 1991; Magull *et al.*,1991), which average 2.68 Å. It should be noted that these types of anions are not common and no reports of the dimeric form presented here are found in the Cambridge Structure Database (Nov 2007 version; Allen, 2002). In this Hg dianion, the doubly bridging selenolates bond to the Hg with bond lengths of 2.6746 (5) Å and 2.8664 (6) Å, while the terminal Hg—Se bond lengths are 2.545 (2), 2.5691 (6) and 2.625 (2) Å for Hg1—Se3B, Hg1—Se1 and Hg1—Se3A, respectively. Packing of the cation/anion pair in this sturcture is consistent with other molecular salts in that there are alternating planes of cations and anions repeated along the crystallographic *c* axis (Fig. 2).

### Experimental

All synthesis were carried out under high purity nitrogen (Welco Praxair) using standard Schlenk and glovebox techniques. Solvents (Aldrich) were purified with a dual column Solv-Tek Solvent Purification System. Lanthanide metals were purchased from Strem. The reported structure, [Yb(DME)_4_]^2+^[Hg_2_(SePh)_6_]^2-^ was synthesized by the reaction of 1 mmol Yb metal with 1.5 mmol PhSeSePh and 40 mg elemental Hg in 30 ml DME. After 14 days, the deep red solution was filtered and concentrated from approximately 20 ml down to 5 ml. The saturated solution was held at -5 C for 7 days to afford yellow crystals of the title compound.

### Refinement

All H atoms were placed in calculated positions with C—H = 0.93–0.97 Å and refined as riding with *U*_iso_(H) = 1.2 *U*_eq_(C) or 1.5 *U*_eq_(C) for methyl H atoms. The Se3/C21—C26 phenylselenolato anion and the C4 methyl carbon atom are disordered over two positions with equal site-occupancy factors of 0.5 and refined using SIMU, SADI and DELU restraints. To improve refinement stability, restraints (DELU and SIMU) were applied to the displacement parameters of all non-H atoms.

### Crystal data

**Table:**

[Yb(C_4_H_10_O_2_)_4_][Hg_2_(C_6_H_5_Se)_6_]	*F*_000_ = 3520
*M**_r_* = 1871.06	*D*_x_ = 2.128 Mg m^−^^3^
Monoclinic, *C*2/*c*	Mo *K*α radiation λ = 0.71073 Å
Hall symbol: -C 2yc	Cell parameters from 7567 reflections
*a* = 22.688 (3) Å	θ = 2.4–30.5º
*b* = 12.354 (2) Å	µ = 10.62 mm^−^^1^
*c* = 22.843 (3) Å	*T* = 100 (2) K
β = 114.218 (3)º	Plate, yellow
*V* = 5839.1 (14) Å^3^	0.17 × 0.14 × 0.02 mm
*Z* = 4	

### Data collection

**Table:**

Bruker SMART APEX CCD diffractometer	5956 independent reflections
Radiation source: fine-focus sealed tube	4806 reflections with *I* > 2σ(*I*)
Monochromator: graphite	*R*_int_ = 0.052
*T* = 100(2) K	θ_max_ = 26.4º
φ and ω scans	θ_min_ = 1.9º
Absorption correction: multi-scan(SADABS; Bruker, 2003)	*h* = −28→28
*T*_min_ = 0.18, *T*_max_ = 0.81	*k* = −15→15
26008 measured reflections	*l* = −28→28

### Refinement

**Table:**

Refinement on *F*^2^	Primary atom site location: structure-invariant direct methods
Least-squares matrix: full	Secondary atom site location: difference Fourier map
*R*[*F*^2^ > 2σ(*F*^2^)] = 0.028	Hydrogen site location: inferred from neighbouring sites
*wR*(*F*^2^) = 0.064	H-atom parameters constrained
*S* = 1.00	*w* = 1/[σ^2^(*F*_o_^2^) + (0.025*P*)^2^ + 0.9*P*] where *P* = (*F*_o_^2^ + 2*F*_c_^2^)/3
5956 reflections	(Δ/σ)_max_ = 0.001
389 parameters	Δρ_max_ = 1.50 e Å^−^^3^
734 restraints	Δρ_min_ = −0.73 e Å^−^^3^
280 constraints	Extinction correction: none

### Special details

**Table:**

Geometry. All e.s.d.'s (except the e.s.d. in the dihedral angle between two l.s. planes) are estimated using the full covariance matrix. The cell e.s.d.'s are taken into account individually in the estimation of e.s.d.'s in distances, angles and torsion angles; correlations between e.s.d.'s in cell parameters are only used when they are defined by crystal symmetry. An approximate (isotropic) treatment of cell e.s.d.'s is used for estimating e.s.d.'s involving l.s. planes.
Refinement. Refinement of *F*^2^ against ALL reflections. The weighted *R*-factor *wR* and goodness of fit *S* are based on *F*^2^, conventional *R*-factors *R* are based on *F*, with *F* set to zero for negative *F*^2^. The threshold expression of *F*^2^ > σ(*F*^2^) is used only for calculating *R*-factors(gt) *etc*. and is not relevant to the choice of reflections for refinement. *R*-factors based on *F*^2^ are statistically about twice as large as those based on *F*, and *R*- factors based on ALL data will be even larger.

### Fractional atomic coordinates and isotropic or equivalent isotropic displacement parameters (Å^2^)

**Table:**

	*x*	*y*	*z*	*U*_iso_*/*U*_eq_	Occ. (<1)
Yb1	0.0000	0.07701 (2)	0.2500	0.02876 (8)	
O1	0.0836 (2)	0.1319 (3)	0.20857 (19)	0.0506 (10)	
O2	−0.0312 (2)	0.2322 (3)	0.1753 (2)	0.0707 (13)	
O3	0.00913 (18)	−0.0821 (3)	0.18739 (19)	0.0439 (9)	
O4	−0.10668 (19)	0.0251 (3)	0.15775 (19)	0.0533 (10)	
C1	0.1380 (3)	0.0671 (6)	0.2132 (3)	0.0624 (17)	
H1A	0.1530	0.0255	0.2521	0.094*	
H1B	0.1722	0.1135	0.2136	0.094*	
H1C	0.1253	0.0192	0.1770	0.094*	
C2	0.0571 (4)	0.1880 (5)	0.1486 (3)	0.0626 (16)	
H2A	0.0339	0.1374	0.1144	0.075*	
H2B	0.0918	0.2194	0.1398	0.075*	
C3	0.0133 (4)	0.2736 (6)	0.1501 (4)	0.0793 (19)	
H3A	0.0380	0.3327	0.1768	0.095*	
H3B	−0.0107	0.3013	0.1071	0.095*	
C4A	−0.0602 (8)	0.3280 (10)	0.1864 (9)	0.061 (4)	0.50
H4A	−0.0897	0.3089	0.2053	0.092*	0.50
H4B	−0.0833	0.3646	0.1464	0.092*	0.50
H4C	−0.0272	0.3748	0.2151	0.092*	0.50
C4B	−0.0863 (6)	0.3006 (13)	0.1677 (10)	0.075 (4)	0.50
H4D	−0.1145	0.2622	0.1823	0.113*	0.50
H4E	−0.1094	0.3191	0.1232	0.113*	0.50
H4F	−0.0713	0.3654	0.1925	0.113*	0.50
C5	0.0553 (3)	−0.1652 (5)	0.2109 (4)	0.070 (2)	
H5A	0.0924	−0.1389	0.2469	0.105*	
H5B	0.0682	−0.1881	0.1777	0.105*	
H5C	0.0369	−0.2254	0.2242	0.105*	
C6	−0.0454 (3)	−0.1146 (5)	0.1329 (3)	0.0596 (16)	
H6A	−0.0664	−0.1746	0.1440	0.072*	
H6B	−0.0319	−0.1392	0.0999	0.072*	
C7	−0.0926 (3)	−0.0226 (5)	0.1071 (3)	0.0565 (15)	
H7A	−0.0745	0.0320	0.0887	0.068*	
H7B	−0.1322	−0.0491	0.0734	0.068*	
C8	−0.1610 (4)	0.0929 (7)	0.1291 (4)	0.091 (3)	
H8A	−0.1694	0.1295	0.1620	0.137*	
H8B	−0.1979	0.0500	0.1035	0.137*	
H8C	−0.1527	0.1453	0.1024	0.137*	
Hg1	0.072118 (8)	0.426875 (14)	0.561656 (9)	0.02549 (6)	
Se1	0.10648 (3)	0.44945 (5)	0.68300 (2)	0.03980 (14)	
Se2	−0.04871 (2)	0.35961 (3)	0.49222 (2)	0.02268 (10)	
C9	0.1760 (2)	0.5511 (4)	0.7052 (2)	0.0340 (11)	
C10	0.2013 (2)	0.5948 (4)	0.7657 (3)	0.0398 (12)	
H10	0.1856	0.5718	0.7954	0.048*	
C11	0.2492 (3)	0.6718 (5)	0.7837 (3)	0.0434 (13)	
H11	0.2654	0.7002	0.8250	0.052*	
C12	0.2731 (2)	0.7066 (5)	0.7412 (3)	0.0505 (14)	
H12	0.3046	0.7603	0.7528	0.061*	
C13	0.2501 (3)	0.6611 (6)	0.6810 (3)	0.0590 (17)	
H13	0.2671	0.6827	0.6521	0.071*	
C14	0.2019 (3)	0.5837 (5)	0.6629 (3)	0.0496 (15)	
H14	0.1868	0.5536	0.6220	0.060*	
C15	−0.0921 (2)	0.3620 (3)	0.5486 (2)	0.0213 (9)	
C16	−0.1494 (2)	0.3053 (4)	0.5311 (2)	0.0330 (11)	
H16	−0.1666	0.2694	0.4919	0.040*	
C17	−0.1812 (2)	0.3015 (5)	0.5714 (3)	0.0439 (13)	
H17	−0.2204	0.2652	0.5583	0.053*	
C18	−0.1556 (2)	0.3506 (4)	0.6302 (2)	0.0342 (11)	
H18	−0.1765	0.3456	0.6576	0.041*	
C19	−0.0986 (2)	0.4075 (4)	0.6484 (2)	0.0291 (10)	
H19	−0.0811	0.4417	0.6881	0.035*	
C20	−0.0677 (2)	0.4141 (4)	0.6079 (2)	0.0281 (10)	
H20	−0.0298	0.4540	0.6203	0.034*	
Se3A	0.14624 (15)	0.36619 (19)	0.50396 (14)	0.0311 (4)	0.50
C21A	0.1583 (3)	0.2179 (5)	0.5311 (4)	0.0246 (18)	0.50
C22A	0.1111 (4)	0.1562 (5)	0.5387 (4)	0.027 (2)	0.50
H22A	0.0721	0.1891	0.5325	0.032*	0.50
C23A	0.1193 (4)	0.0471 (5)	0.5553 (4)	0.0345 (19)	0.50
H23A	0.0866	0.0076	0.5601	0.041*	0.50
C24A	0.1772 (4)	−0.0007 (6)	0.5643 (4)	0.036 (2)	0.50
H24A	0.1836	−0.0736	0.5753	0.044*	0.50
C25A	0.2267 (4)	0.0574 (6)	0.5572 (4)	0.036 (2)	0.50
H25A	0.2656	0.0242	0.5634	0.043*	0.50
C26A	0.2163 (3)	0.1663 (6)	0.5406 (4)	0.0273 (18)	0.50
H26A	0.2489	0.2059	0.5358	0.033*	0.50
Se3B	0.14874 (15)	0.3380 (2)	0.52023 (15)	0.0452 (6)	0.50
C21B	0.1398 (4)	0.1869 (5)	0.5340 (4)	0.034 (2)	0.50
C22B	0.0823 (4)	0.1404 (6)	0.5287 (4)	0.039 (2)	0.50
H22B	0.0465	0.1849	0.5196	0.047*	0.50
C23B	0.0758 (4)	0.0299 (6)	0.5365 (4)	0.048 (2)	0.50
H23B	0.0366	0.0005	0.5327	0.057*	0.50
C24B	0.1294 (5)	−0.0345 (6)	0.5500 (4)	0.055 (2)	0.50
H24B	0.1259	−0.1085	0.5553	0.066*	0.50
C25B	0.1886 (5)	0.0073 (7)	0.5559 (5)	0.050 (2)	0.50
H25B	0.2242	−0.0375	0.5650	0.059*	0.50
C26B	0.1928 (4)	0.1183 (7)	0.5477 (4)	0.046 (2)	0.50
H26B	0.2319	0.1475	0.5515	0.055*	0.50

### Atomic displacement parameters (Å^2^)

**Table:**

	*U*^11^	*U*^22^	*U*^33^	*U*^12^	*U*^13^	*U*^23^
Yb1	0.05749 (19)	0.01481 (14)	0.01899 (16)	0.000	0.02075 (14)	0.000
O1	0.083 (3)	0.044 (2)	0.039 (2)	−0.0086 (18)	0.040 (2)	0.0020 (18)
O2	0.130 (3)	0.037 (2)	0.076 (3)	0.029 (2)	0.073 (3)	0.028 (2)
O3	0.053 (2)	0.0304 (18)	0.042 (2)	0.0060 (15)	0.0138 (18)	−0.0135 (16)
O4	0.059 (2)	0.064 (2)	0.032 (2)	0.0202 (19)	0.0129 (19)	0.0095 (19)
C1	0.069 (4)	0.083 (5)	0.043 (4)	−0.006 (3)	0.030 (3)	−0.005 (3)
C2	0.107 (4)	0.056 (4)	0.049 (3)	0.000 (3)	0.057 (3)	0.009 (3)
C3	0.132 (5)	0.057 (4)	0.074 (4)	0.013 (3)	0.068 (4)	0.030 (3)
C4A	0.110 (9)	0.033 (6)	0.050 (8)	0.025 (6)	0.041 (7)	0.011 (5)
C4B	0.108 (8)	0.042 (7)	0.072 (10)	0.033 (6)	0.033 (7)	0.002 (6)
C5	0.066 (4)	0.051 (4)	0.079 (5)	0.012 (3)	0.016 (3)	−0.019 (3)
C6	0.070 (4)	0.050 (3)	0.047 (4)	0.000 (3)	0.011 (3)	−0.014 (3)
C7	0.073 (4)	0.056 (3)	0.034 (3)	0.004 (3)	0.016 (3)	−0.007 (3)
C8	0.082 (4)	0.125 (6)	0.057 (5)	0.058 (4)	0.020 (4)	0.025 (4)
Hg1	0.02478 (9)	0.02907 (10)	0.02306 (11)	0.00644 (7)	0.01028 (8)	0.00076 (8)
Se1	0.0472 (3)	0.0482 (3)	0.0198 (3)	−0.0124 (2)	0.0094 (2)	0.0091 (2)
Se2	0.0279 (2)	0.0224 (2)	0.0226 (3)	−0.00359 (17)	0.0153 (2)	−0.00224 (19)
C9	0.026 (2)	0.047 (3)	0.023 (2)	0.0002 (19)	0.0041 (19)	0.006 (2)
C10	0.040 (3)	0.051 (3)	0.027 (3)	−0.004 (2)	0.013 (2)	0.004 (2)
C11	0.043 (3)	0.056 (3)	0.026 (3)	−0.006 (2)	0.009 (2)	−0.007 (2)
C12	0.031 (3)	0.071 (4)	0.040 (3)	−0.017 (3)	0.005 (2)	0.000 (3)
C13	0.045 (3)	0.099 (5)	0.030 (3)	−0.031 (3)	0.014 (3)	−0.001 (3)
C14	0.037 (3)	0.084 (4)	0.026 (3)	−0.020 (3)	0.010 (2)	−0.002 (3)
C15	0.025 (2)	0.019 (2)	0.025 (2)	0.0055 (16)	0.0151 (19)	0.0036 (18)
C16	0.025 (2)	0.051 (3)	0.026 (3)	−0.002 (2)	0.013 (2)	−0.006 (2)
C17	0.023 (2)	0.076 (4)	0.033 (3)	−0.013 (2)	0.013 (2)	−0.010 (3)
C18	0.029 (2)	0.051 (3)	0.029 (3)	0.002 (2)	0.018 (2)	−0.002 (2)
C19	0.041 (2)	0.025 (2)	0.024 (3)	−0.0010 (19)	0.016 (2)	−0.0046 (19)
C20	0.034 (2)	0.025 (2)	0.030 (3)	−0.0068 (18)	0.017 (2)	−0.0047 (19)
Se3A	0.0342 (7)	0.0292 (10)	0.0414 (11)	0.0113 (7)	0.0273 (7)	0.0129 (7)
C21A	0.028 (4)	0.029 (3)	0.020 (4)	0.003 (3)	0.013 (3)	−0.001 (3)
C22A	0.028 (4)	0.028 (4)	0.024 (4)	0.006 (3)	0.011 (4)	0.004 (4)
C23A	0.050 (4)	0.029 (4)	0.029 (4)	0.009 (3)	0.022 (4)	0.003 (3)
C24A	0.054 (4)	0.032 (4)	0.026 (4)	0.016 (3)	0.019 (4)	0.006 (4)
C25A	0.040 (4)	0.035 (4)	0.029 (4)	0.018 (3)	0.011 (4)	0.000 (4)
C26A	0.027 (4)	0.034 (4)	0.020 (4)	0.008 (3)	0.010 (3)	0.002 (4)
Se3B	0.0400 (9)	0.0440 (14)	0.069 (2)	0.0054 (9)	0.0405 (13)	−0.0030 (10)
C21B	0.041 (5)	0.038 (4)	0.020 (4)	0.015 (3)	0.010 (4)	−0.003 (4)
C22B	0.055 (5)	0.039 (4)	0.026 (5)	0.008 (4)	0.019 (5)	0.007 (4)
C23B	0.068 (5)	0.044 (4)	0.036 (5)	0.011 (4)	0.026 (5)	0.006 (4)
C24B	0.082 (5)	0.050 (5)	0.036 (5)	0.022 (4)	0.027 (5)	0.007 (4)
C25B	0.066 (4)	0.048 (4)	0.031 (5)	0.032 (4)	0.017 (4)	−0.003 (4)
C26B	0.052 (4)	0.051 (4)	0.029 (4)	0.017 (3)	0.009 (4)	−0.009 (4)

### Geometric parameters (Å, °)

**Table:**

Yb1—O2	2.469 (4)	Se2—C15	1.917 (4)
Yb1—O2^i^	2.469 (4)	C9—C10	1.372 (7)
Yb1—O3	2.489 (3)	C9—C14	1.381 (7)
Yb1—O3^i^	2.489 (3)	C10—C11	1.374 (7)
Yb1—O1	2.534 (4)	C10—H10	0.9300
Yb1—O1^i^	2.534 (4)	C11—C12	1.360 (8)
Yb1—O4^i^	2.554 (4)	C11—H11	0.9300
Yb1—O4	2.555 (4)	C12—C13	1.375 (8)
O1—C2	1.430 (7)	C12—H12	0.9300
O1—C1	1.439 (7)	C13—C14	1.382 (8)
O2—C4A	1.427 (8)	C13—H13	0.9300
O2—C3	1.445 (7)	C14—H14	0.9300
O2—C4B	1.459 (9)	C15—C16	1.383 (6)
O3—C5	1.407 (7)	C15—C20	1.392 (6)
O3—C6	1.407 (7)	C16—C17	1.383 (7)
O4—C8	1.410 (7)	C16—H16	0.9300
O4—C7	1.447 (7)	C17—C18	1.368 (7)
C1—H1A	0.9600	C17—H17	0.9300
C1—H1B	0.9600	C18—C19	1.378 (6)
C1—H1C	0.9600	C18—H18	0.9300
C2—C3	1.462 (9)	C19—C20	1.372 (6)
C2—H2A	0.9700	C19—H19	0.9300
C2—H2B	0.9700	C20—H20	0.9300
C3—H3A	0.9700	Se3A—C21A	1.917 (5)
C3—H3B	0.9700	C21A—C22A	1.384 (6)
C4A—H4A	0.9600	C21A—C26A	1.396 (6)
C4A—H4B	0.9600	C22A—C23A	1.392 (6)
C4A—H4C	0.9600	C22A—H22A	0.9300
C4B—H4D	0.9600	C23A—C24A	1.377 (6)
C4B—H4E	0.9600	C23A—H23A	0.9300
C4B—H4F	0.9600	C24A—C25A	1.396 (7)
C5—H5A	0.9600	C24A—H24A	0.9300
C5—H5B	0.9600	C25A—C26A	1.392 (6)
C5—H5C	0.9600	C25A—H25A	0.9300
C6—C7	1.506 (8)	C26A—H26A	0.9300
C6—H6A	0.9700	Se3B—C21B	1.917 (6)
C6—H6B	0.9700	C21B—C22B	1.386 (6)
C7—H7A	0.9700	C21B—C26B	1.396 (6)
C7—H7B	0.9700	C22B—C23B	1.393 (6)
C8—H8A	0.9600	C22B—H22B	0.9300
C8—H8B	0.9600	C23B—C24B	1.379 (6)
C8—H8C	0.9600	C23B—H23B	0.9300
Hg1—Se3B	2.544 (3)	C24B—C25B	1.393 (7)
Hg1—Se1	2.5683 (7)	C24B—H24B	0.9300
Hg1—Se3A	2.635 (3)	C25B—C26B	1.392 (7)
Hg1—Se2	2.6747 (6)	C25B—H25B	0.9300
Hg1—Se2^ii^	2.8667 (6)	C26B—H26B	0.9300
Se1—C9	1.914 (5)		
			
O2—Yb1—O2^i^	78.1 (2)	H8A—C8—H8B	109.5
O2—Yb1—O3	106.50 (14)	O4—C8—H8C	109.5
O2^i^—Yb1—O3	160.44 (15)	H8A—C8—H8C	109.5
O2—Yb1—O3^i^	160.44 (15)	H8B—C8—H8C	109.5
O2^i^—Yb1—O3^i^	106.50 (14)	Se3B—Hg1—Se1	119.81 (7)
O3—Yb1—O3^i^	75.68 (18)	Se3B—Hg1—Se3A	10.79 (6)
O2—Yb1—O1	65.21 (14)	Se1—Hg1—Se3A	126.67 (7)
O2^i^—Yb1—O1	90.22 (13)	Se3B—Hg1—Se2	109.62 (8)
O3—Yb1—O1	75.25 (13)	Se1—Hg1—Se2	117.211 (17)
O3^i^—Yb1—O1	132.63 (13)	Se3A—Hg1—Se2	108.91 (8)
O2—Yb1—O1^i^	90.22 (13)	Se3B—Hg1—Se2^ii^	105.78 (5)
O2^i^—Yb1—O1^i^	65.21 (14)	Se1—Hg1—Se2^ii^	106.512 (17)
O3—Yb1—O1^i^	132.63 (13)	Se3A—Hg1—Se2^ii^	95.21 (4)
O3^i^—Yb1—O1^i^	75.25 (13)	Se2—Hg1—Se2^ii^	93.839 (14)
O1—Yb1—O1^i^	148.96 (18)	C9—Se1—Hg1	102.10 (15)
O2—Yb1—O4^i^	132.16 (15)	C15—Se2—Hg1	106.31 (13)
O2^i^—Yb1—O4^i^	73.67 (16)	C15—Se2—Hg1^ii^	102.62 (12)
O3—Yb1—O4^i^	90.09 (13)	Hg1—Se2—Hg1^ii^	86.163 (14)
O3^i^—Yb1—O4^i^	66.53 (12)	C10—C9—C14	117.9 (5)
O1—Yb1—O4^i^	76.96 (13)	C10—C9—Se1	119.0 (4)
O1^i^—Yb1—O4^i^	111.10 (13)	C14—C9—Se1	123.1 (4)
O2—Yb1—O4	73.66 (16)	C9—C10—C11	121.7 (5)
O2^i^—Yb1—O4	132.16 (15)	C9—C10—H10	119.1
O3—Yb1—O4	66.53 (12)	C11—C10—H10	119.1
O3^i^—Yb1—O4	90.09 (13)	C12—C11—C10	120.3 (5)
O1—Yb1—O4	111.10 (13)	C12—C11—H11	119.9
O1^i^—Yb1—O4	76.96 (13)	C10—C11—H11	119.9
O4^i^—Yb1—O4	150.91 (18)	C11—C12—C13	119.0 (5)
C2—O1—C1	110.5 (4)	C11—C12—H12	120.5
C2—O1—Yb1	113.8 (4)	C13—C12—H12	120.5
C1—O1—Yb1	125.1 (3)	C12—C13—C14	120.8 (5)
C4A—O2—C3	103.0 (8)	C12—C13—H13	119.6
C3—O2—C4B	116.8 (10)	C14—C13—H13	119.6
C4A—O2—Yb1	122.9 (8)	C9—C14—C13	120.3 (6)
C3—O2—Yb1	120.1 (4)	C9—C14—H14	119.9
C4B—O2—Yb1	121.4 (10)	C13—C14—H14	119.9
C5—O3—C6	112.1 (4)	C16—C15—C20	117.8 (4)
C5—O3—Yb1	125.5 (4)	C16—C15—Se2	118.6 (3)
C6—O3—Yb1	118.9 (3)	C20—C15—Se2	123.5 (3)
C8—O4—C7	108.0 (5)	C15—C16—C17	120.6 (5)
C8—O4—Yb1	125.9 (5)	C15—C16—H16	119.7
C7—O4—Yb1	108.5 (3)	C17—C16—H16	119.7
O1—C1—H1A	109.5	C18—C17—C16	120.8 (5)
O1—C1—H1B	109.5	C18—C17—H17	119.6
H1A—C1—H1B	109.5	C16—C17—H17	119.6
O1—C1—H1C	109.5	C17—C18—C19	119.3 (4)
H1A—C1—H1C	109.5	C17—C18—H18	120.3
H1B—C1—H1C	109.5	C19—C18—H18	120.3
O1—C2—C3	110.5 (5)	C20—C19—C18	120.2 (5)
O1—C2—H2A	109.6	C20—C19—H19	119.9
C3—C2—H2A	109.6	C18—C19—H19	119.9
O1—C2—H2B	109.6	C19—C20—C15	121.3 (4)
C3—C2—H2B	109.6	C19—C20—H20	119.4
H2A—C2—H2B	108.1	C15—C20—H20	119.4
O2—C3—C2	110.3 (5)	C21A—Se3A—Hg1	98.4 (2)
O2—C3—H3A	109.6	C22A—C21A—C26A	117.2 (6)
C2—C3—H3A	109.6	C22A—C21A—Se3A	123.5 (5)
O2—C3—H3B	109.6	C26A—C21A—Se3A	119.2 (5)
C2—C3—H3B	109.6	C21A—C22A—C23A	122.9 (6)
H3A—C3—H3B	108.1	C21A—C22A—H22A	118.5
O2—C4A—H4A	109.5	C23A—C22A—H22A	118.5
O2—C4A—H4B	109.5	C24A—C23A—C22A	118.0 (7)
H4A—C4A—H4B	109.5	C24A—C23A—H23A	121.0
O2—C4A—H4C	109.5	C22A—C23A—H23A	121.0
H4A—C4A—H4C	109.5	C23A—C24A—C25A	121.7 (7)
H4B—C4A—H4C	109.5	C23A—C24A—H24A	119.1
O2—C4B—H4D	109.5	C25A—C24A—H24A	119.1
O2—C4B—H4E	109.5	C26A—C25A—C24A	118.3 (7)
H4D—C4B—H4E	109.5	C26A—C25A—H25A	120.9
O2—C4B—H4F	109.5	C24A—C25A—H25A	120.9
H4D—C4B—H4F	109.5	C25A—C26A—C21A	121.9 (6)
H4E—C4B—H4F	109.5	C25A—C26A—H26A	119.1
O3—C5—H5A	109.5	C21A—C26A—H26A	119.1
O3—C5—H5B	109.5	C21B—Se3B—Hg1	103.0 (3)
H5A—C5—H5B	109.5	C22B—C21B—C26B	117.4 (6)
O3—C5—H5C	109.5	C22B—C21B—Se3B	123.2 (5)
H5A—C5—H5C	109.5	C26B—C21B—Se3B	119.3 (6)
H5B—C5—H5C	109.5	C21B—C22B—C23B	122.7 (7)
O3—C6—C7	110.9 (5)	C21B—C22B—H22B	118.6
O3—C6—H6A	109.5	C23B—C22B—H22B	118.6
C7—C6—H6A	109.5	C24B—C23B—C22B	117.7 (7)
O3—C6—H6B	109.5	C24B—C23B—H23B	121.2
C7—C6—H6B	109.5	C22B—C23B—H23B	121.2
H6A—C6—H6B	108.0	C23B—C24B—C25B	122.3 (7)
O4—C7—C6	110.5 (5)	C23B—C24B—H24B	118.8
O4—C7—H7A	109.6	C25B—C24B—H24B	118.8
C6—C7—H7A	109.6	C26B—C25B—C24B	118.0 (7)
O4—C7—H7B	109.6	C26B—C25B—H25B	121.0
C6—C7—H7B	109.6	C24B—C25B—H25B	121.0
H7A—C7—H7B	108.1	C25B—C26B—C21B	121.9 (7)
O4—C8—H8A	109.5	C25B—C26B—H26B	119.1
O4—C8—H8B	109.5	C21B—C26B—H26B	119.1
			
O2—Yb1—O1—C2	−24.1 (4)	O1—C2—C3—O2	−48.0 (8)
O2^i^—Yb1—O1—C2	−100.9 (4)	C5—O3—C6—C7	−177.5 (5)
O3—Yb1—O1—C2	92.3 (4)	Yb1—O3—C6—C7	22.1 (7)
O3^i^—Yb1—O1—C2	146.2 (4)	C8—O4—C7—C6	−166.9 (6)
O1^i^—Yb1—O1—C2	−64.7 (4)	Yb1—O4—C7—C6	53.8 (6)
O4^i^—Yb1—O1—C2	−174.1 (4)	O3—C6—C7—O4	−51.4 (7)
O4—Yb1—O1—C2	35.2 (4)	Se3B—Hg1—Se1—C9	66.62 (17)
O2—Yb1—O1—C1	−165.2 (5)	Se3A—Hg1—Se1—C9	56.65 (17)
O2^i^—Yb1—O1—C1	118.0 (5)	Se2—Hg1—Se1—C9	−156.51 (15)
O3—Yb1—O1—C1	−48.7 (4)	Se2^ii^—Hg1—Se1—C9	−53.16 (15)
O3^i^—Yb1—O1—C1	5.1 (5)	Se3B—Hg1—Se2—C15	149.70 (14)
O1^i^—Yb1—O1—C1	154.2 (5)	Se1—Hg1—Se2—C15	8.74 (13)
O4^i^—Yb1—O1—C1	44.9 (4)	Se3A—Hg1—Se2—C15	161.11 (13)
O4—Yb1—O1—C1	−105.9 (4)	Se2^ii^—Hg1—Se2—C15	−102.04 (13)
O2^i^—Yb1—O2—C4A	−39.6 (8)	Se3B—Hg1—Se2—Hg1^ii^	−108.26 (5)
O3—Yb1—O2—C4A	160.0 (9)	Se1—Hg1—Se2—Hg1^ii^	110.779 (18)
O3^i^—Yb1—O2—C4A	66.4 (10)	Se3A—Hg1—Se2—Hg1^ii^	−96.85 (4)
O1—Yb1—O2—C4A	−135.4 (9)	Se2^ii^—Hg1—Se2—Hg1^ii^	0.0
O1^i^—Yb1—O2—C4A	25.0 (9)	Hg1—Se1—C9—C10	171.1 (4)
O4^i^—Yb1—O2—C4A	−94.3 (9)	Hg1—Se1—C9—C14	−8.3 (5)
O4—Yb1—O2—C4A	101.3 (9)	C14—C9—C10—C11	2.4 (8)
O2^i^—Yb1—O2—C3	93.7 (5)	Se1—C9—C10—C11	−177.0 (4)
O3—Yb1—O2—C3	−66.7 (5)	C9—C10—C11—C12	−0.2 (9)
O3^i^—Yb1—O2—C3	−160.3 (5)	C10—C11—C12—C13	−2.1 (9)
O1—Yb1—O2—C3	−2.2 (5)	C11—C12—C13—C14	2.1 (10)
O1^i^—Yb1—O2—C3	158.2 (5)	C10—C9—C14—C13	−2.4 (9)
O4^i^—Yb1—O2—C3	39.0 (5)	Se1—C9—C14—C13	177.1 (5)
O4—Yb1—O2—C3	−125.4 (5)	C12—C13—C14—C9	0.1 (10)
O2^i^—Yb1—O2—C4B	−71.0 (9)	Hg1—Se2—C15—C16	−163.3 (3)
O3—Yb1—O2—C4B	128.6 (9)	Hg1^ii^—Se2—C15—C16	107.0 (3)
O3^i^—Yb1—O2—C4B	35.0 (10)	Hg1—Se2—C15—C20	13.8 (4)
O1—Yb1—O2—C4B	−166.9 (9)	Hg1^ii^—Se2—C15—C20	−75.9 (4)
O1^i^—Yb1—O2—C4B	−6.5 (9)	C20—C15—C16—C17	0.4 (7)
O4^i^—Yb1—O2—C4B	−125.7 (9)	Se2—C15—C16—C17	177.7 (4)
O4—Yb1—O2—C4B	69.9 (9)	C15—C16—C17—C18	−2.2 (8)
O2—Yb1—O3—C5	143.0 (5)	C16—C17—C18—C19	2.2 (8)
O2^i^—Yb1—O3—C5	41.9 (7)	C17—C18—C19—C20	−0.5 (8)
O3^i^—Yb1—O3—C5	−57.2 (4)	C18—C19—C20—C15	−1.3 (7)
O1—Yb1—O3—C5	85.0 (5)	C16—C15—C20—C19	1.3 (7)
O1^i^—Yb1—O3—C5	−110.9 (5)	Se2—C15—C20—C19	−175.8 (4)
O4^i^—Yb1—O3—C5	8.5 (5)	Se3B—Hg1—Se3A—C21A	16.5 (7)
O4—Yb1—O3—C5	−153.6 (5)	Se1—Hg1—Se3A—C21A	70.0 (3)
O2—Yb1—O3—C6	−59.5 (4)	Se2—Hg1—Se3A—C21A	−79.1 (3)
O2^i^—Yb1—O3—C6	−160.6 (5)	Se2^ii^—Hg1—Se3A—C21A	−175.0 (3)
O3^i^—Yb1—O3—C6	100.4 (4)	Hg1—Se3A—C21A—C22A	35.3 (5)
O1—Yb1—O3—C6	−117.4 (4)	Hg1—Se3A—C21A—C26A	−148.2 (4)
O1^i^—Yb1—O3—C6	46.7 (5)	C26A—C21A—C22A—C23A	0.00 (3)
O4^i^—Yb1—O3—C6	166.1 (4)	Se3A—C21A—C22A—C23A	176.6 (6)
O4—Yb1—O3—C6	3.9 (4)	C21A—C22A—C23A—C24A	0.00 (3)
O2—Yb1—O4—C8	−43.4 (5)	C22A—C23A—C24A—C25A	0.00 (7)
O2^i^—Yb1—O4—C8	13.0 (6)	C23A—C24A—C25A—C26A	−0.01 (9)
O3—Yb1—O4—C8	−160.1 (5)	C24A—C25A—C26A—C21A	0.01 (9)
O3^i^—Yb1—O4—C8	125.6 (5)	C22A—C21A—C26A—C25A	−0.01 (7)
O1—Yb1—O4—C8	−97.8 (5)	Se3A—C21A—C26A—C25A	−176.8 (6)
O1^i^—Yb1—O4—C8	50.8 (5)	Se1—Hg1—Se3B—C21B	77.9 (3)
O4^i^—Yb1—O4—C8	160.9 (5)	Se3A—Hg1—Se3B—C21B	−150.0 (10)
O2—Yb1—O4—C7	86.7 (4)	Se2—Hg1—Se3B—C21B	−61.9 (3)
O2^i^—Yb1—O4—C7	143.0 (3)	Se2^ii^—Hg1—Se3B—C21B	−162.0 (3)
O3—Yb1—O4—C7	−30.1 (3)	Hg1—Se3B—C21B—C22B	34.0 (5)
O3^i^—Yb1—O4—C7	−104.4 (4)	Hg1—Se3B—C21B—C26B	−148.4 (4)
O1—Yb1—O4—C7	32.2 (4)	C26B—C21B—C22B—C23B	0.00 (3)
O1^i^—Yb1—O4—C7	−179.2 (4)	Se3B—C21B—C22B—C23B	177.6 (7)
O4^i^—Yb1—O4—C7	−69.1 (3)	C21B—C22B—C23B—C24B	0.00 (3)
C1—O1—C2—C3	−165.6 (6)	C22B—C23B—C24B—C25B	0.00 (7)
Yb1—O1—C2—C3	47.6 (7)	C23B—C24B—C25B—C26B	0.00 (9)
C4A—O2—C3—C2	168.1 (9)	C24B—C25B—C26B—C21B	0.01 (9)
C4B—O2—C3—C2	−167.7 (10)	C22B—C21B—C26B—C25B	−0.01 (7)
Yb1—O2—C3—C2	26.9 (8)	Se3B—C21B—C26B—C25B	−177.7 (6)

Symmetry codes: (i) −*x*, *y*, −*z*+1/2; (ii) −*x*, −*y*+1, −*z*+1.
